# Fast and Accurate Prediction of Light Scattering from Plasmonic Nanoarrays in Multiple Directions

**DOI:** 10.3390/mi13040613

**Published:** 2022-04-14

**Authors:** Ting Wan, Tianhao Chen, Yang Bao, Shiyi Wang

**Affiliations:** 1 Department of Communication Engineering, Nanjing University of Posts and Telecommunications, Nanjing 210003, China; 1319015112@njupt.edu.cn (T.C.); brianbao@njupt.edu.cn (Y.B.); B18010213@njupt.edu.cn (S.W.); 2 State Key Laboratory of Millimeter Waves, Nanjing 210096, China

**Keywords:** light scattering, numerical prediction, multiple directions, method of moments, plasmonic nanoarray

## Abstract

The method of moments (MoM) is an efficient electromagnetic numerical method for the accurate prediction of light scattering from plasmonic nanostructures. In practice, the light-scattering properties in different incident directions are often concerning. However, traditional MoM generally resorts to the iterative method, which suffers from the problems of convergence rate and redundant computations for multiple incident excitations. Nanoarray structures will further aggravate these problems due to a large number of unknowns. In this article, an efficient numerical method based on MoM and a hierarchical matrix (H-matrix) algorithm is proposed to solve these problems. Numerical experiments demonstrate the efficiency and accuracy of the proposed method for the prediction of light scattering from plasmonic nanoarrays in multiple directions.

## 1. Introduction

Metal nanoparticles have unique optical properties. When incident light interacts with metal nanoparticles, the absorption and scattering of incident light can be greatly enhanced, which results in the effect of localized surface plasmon resonance (LSPR) [1,2,3,4,5,6,7]. The LSPR is affected by the angle of the incident light, particle shape, material, etc. In medicine, the enhanced energy can be converted into heat to kill cancer cells [8], while it can significantly improve the absorption efficiency of solar cells in the field of photovoltaic energy [9,10,11,12]. Therefore, the research on light scattering from plasmonic nanoparticles has broad prospects. The actual effect of these applications is closely related to the light-scattering parameters, such as the extinction cross section (ECS). In practice, for a given nanostructure, it is often necessary to calculate the ECSs at many different incident angles to describe the optical properties. How to efficiently predict the ECS under multiple incident directions is the motivation of this research work.

Electromagnetic numerical methods play an important role in the prediction and analysis of light scattering because of their high accuracy and good ability to deal with complex structures. The finite element method (FEM) and method of moments (MoM) are the two most commonly used numerical methods in computational electromagnetics. FEM is based on differential equations, which are easy to understand and highly adaptable. However, it introduces additional space and adopts volume discretization, which leads to a large number of unknowns and low efficiency [13,14]. MoM is an integral equation method based on Green’s function, which naturally satisfies the Sommerfeld radiation boundary condition. Hence, it is very suitable for open-domain scattering problems, such as the analysis of the extinction characteristics of nanostructures [15,16,17]. Compared with the FEM, the accuracy of the MoM is higher because there is no numerical dispersion error. Besides, the MoM only needs surface discretization, which significantly reduces the number of unknowns. In summary, compared with the FEM, the MoM is more suitable for analyzing the light scattering because of the smaller number of unknowns and the higher solution accuracy. Therefore, we research light scattering based on the MoM framework.

The iterative method and direct method can be employed to solve the matrix equations of the MoM. For large-scale problems, the iterative method is usually adopted because of the low computational complexity of its key operations of matrix-vector products. However, when the behavior of the system matrix is poor, the iteration converges very slowly and even cannot converge. Furthermore, when dealing with multiple right-hand-side problems, such as the cases of light scattering in multiple directions, the iterative process needs to be repeated for each direction, which is very inefficient [18,19,20,21]. The direct method can circumvent these obstacles, but it is often infeasible due to its high computational costs when dealing with the dense MoM matrix. In this paper, a numerical algorithm based on the hierarchical matrix (H-matrix) method is proposed to construct an efficient direct method for the MoM. It uses a data-sparse format to efficiently approximate the dense matrix of the MoM and solves the matrix equation by using H-LU decomposition algorithm. With the proposed method, the H-LU decomposition only needs to be performed once and stored, then the results can be obtained for each incident light very quickly using H-matrix formatted substitutions. Hence, the proposed method is very suitable for the analysis of light scattering from multiple incident directions. The proposed method can deal with the plasmonic nanostructures with arbitrary shape and arbitrary homogeneous material. For complex inhomogeneous materials, the MoM should be extended or adopts the volume integral equation. Numerical examples in this paper compare the proposed method with traditional MoM, and the results demonstrate its accuracy and efficiency.

## 2. Methods

### 2.1. Method of Moments

The implementation of MoM includes: (1) dividing the object by triangular patches; (2) choosing appropriate basis functions; (3) generating the impedance matrix using Galerkin’s method; (4) solving the matrix equation to obtain the current coefficients. Since the plasmonic metal materials behave as a homogeneous dielectric property at optical frequencies, we adopt the MoM method based on the electric current and magnetic current combined field integral equation (JMCFIE) as follows.

**Figure 1:**
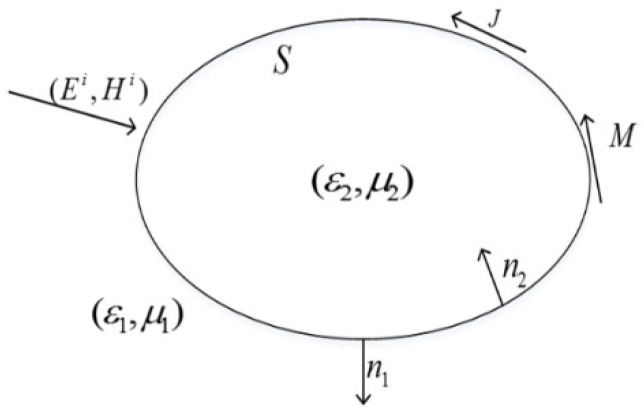
Light scattering from a plasmonic metal object.

As shown in Figure 1, the permittivity and permeability of the medium in the outer space are $\varepsilon_{1}$, $\mu_{1}$, the medium parameters of the object are $\varepsilon_{2}$, $\mu_{2}$. $n_{1}$ and $n_{2}$ represent the normal vector pointing to the outside and inside of the object surface, respectively. We can then obtain the electric-field-integral equation, *EFIE*_1_, and the magnetic-field-integral equation, *MFIE*_1_, outside the medium as follows [22]:

(1) $$-M_{1}-n_{1}\times \left[\eta_{1}L_{1}\left(J_{1}\right)-K_{1}(M_{1})\right]=n_{1}\times E^{i}$$

(2) $$J_{1}-n_{1}\times \left[\frac{1}{\eta_{1}}L_{1}\left(M_{1}\right)-K_{1}(J_{1})\right]=n_{1}\times H^{i}$$

The *EFIE*_2_ and the *MFIE*_2_ inside the medium can also be obtained as follows:

(3) $$-M_{2}=n_{2}\times \left[\eta_{2}L_{2}\left(J_{2}\right)-K_{2}(M_{2})\right]$$

(4) $$J_{2}=n_{2}\times \left[\frac{1}{\eta_{2}}L_{2}\left(M_{2}\right)+K_{2}(J_{2})\right]$$

where *J* is the equivalent current, *M* is the equivalent magnetic current, and *L* and *K* are integral operators. The combined field integral equation (CFIE) is given by:

(5) $$a_{l}EFIE_{l}+b_{l}\eta_{l}n_{l}\times MFIE_{l}$$

Here, $a_{l}=\alpha$, $b_{l}=(1-\alpha )$, 0<α<1, $\eta_{l}=\sqrt{\mu_{l}/\varepsilon_{l}}$, l=1 or 2. It can be seen that Equation (5) denotes a combination of Equations (1) and (2), or Equations (3) and (4).

The linear combination of *EFIE* and *MFIE* can generate different forms of *CFIE*, in which the electric current CFIE (*JCFIE*) and the magnetic current CFIE (*MCFIE*) can be written as:

(6) $$JCFIE_{l}:\alpha_{l}EFIE_{l}+\beta_{l}\eta_{l}n_{l}\times MFIE_{l}$$

(7) $$MCFIE_{l}:\alpha_{l}\eta_{l}MFIE_{l}-\beta_{l}n_{l}\times EFIE_{l}$$

Then, the *JMCFIE* is obtained by combining the above equations as follows:

(8) $$\left\{ \begin{array}{l} JCFIE_{1}+JCFIE_{2}\\ MCFIE_{1}+MCFIE_{2} \end{array}\right.$$

By introducing RWG basis functions to expand *J* and *M*, and employing Galerkin’s approach discrete the *JMCFIE* equation, the resulting MoM matrix equation can be obtained as

(9) [Z]·{I}={b}

where *Z* denotes the system matrix of the MoM, *I* denotes the vector of unknown current coefficients, and *b* represents the right-hand-side (RHS) vector, which is related to the incident light.

### 2.2. H-Matrix Method

**Figure 2:**
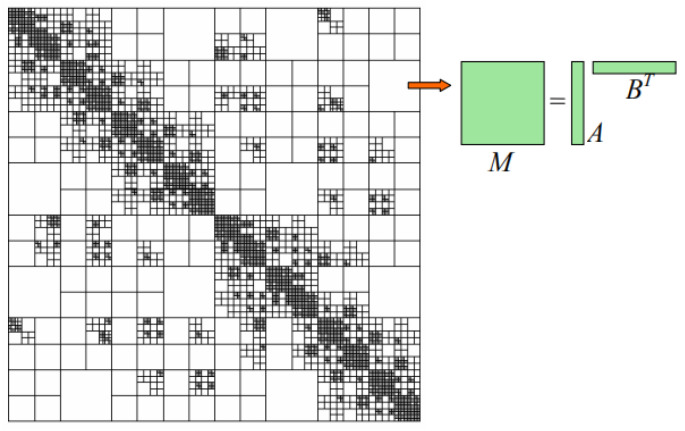
A schematic diagram of a typical H-matrix structure. Black represents full matrix blocks and white represents low-rank matrix blocks.

**Figure 3:**
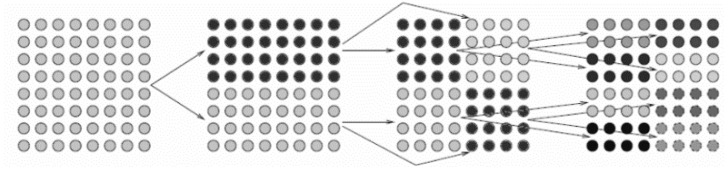
Recursive bisection group tree structure of index set.

Figure 2 shows a schematic diagram of a typical H-matrix structure. The key idea of the H-matrix method is to approximate a dense matrix M into a data-sparse matrix ABT. Based on this, one can construct an H-matrix representation of the MoM system matrix Z in (9) and develop an efficient H-matrix-based direct method to solve (9). The construction of an H-matrix can be described by the following steps:

First, a cluster tree should be built. A cluster is a finite indexed set of basis functions. We define a primitive cluster *I* = {1,2,…,N} to represent all basis functions. A cluster tree *T_I_* is generated by a recursive subdivision of I. One index set is subdivided into two subsets recursively until the number of basis functions in the subset (denoted as “#”) is smaller than a threshold *n*_leaf_. The resulting cluster tree is called a binary tree, as shown in Figure 3.

Then, the block cluster tree $T_{I\times I}$ can be constructed by the hierarchical division of I×I. A block cluster tree is nothing but the interaction of two cluster trees: $T_{I}$ of the original-basis function set and $T_{I}$ of the testing-basis function set. The block cluster tree terminates at blocks $t\times s\in T_{I\times I}$ ($t\in T_{I}$ and $s\in T_{J}$), satisfying:

1. $\#t\leq n_{\mathrm{leaf}}$ or $\#s\leq n_{\mathrm{leaf}}$
2. Clusters *t* and *s* satisfy the admissibility condition of
  (10) $$\min\left\{diam\left(\Omega_{t}\right),diam\left(\Omega_{s}\right)\right\}\leq \eta dist\left(\Omega_{t},\Omega_{s}\right)$$
  where *diam* and *dist* denote the Euclidean diameter and distance of the supports of the basis functions in *s*, *t*, and η>0 controls the trade-off between admissible blocks. Blocks $t\times s\in T_{I\times I}$ satisfying (10) are called admissible blocks, which can be approximated by low-rank matrices in the following representation:
  (11) $$G=XY^{T}\left(G\in {\mathbb{R}}^{m\times n},X\in {\mathbb{R}}^{m\times k},Y\in {\mathbb{R}}^{n\times k},k\ll m,n\right)$$

There are only two types of blocks in $T_{I\times I}$, i.e., admissible blocks stored as low-rank matrices and inadmissible blocks stored as full matrices.

To construct the H-matrix *Z_H_*, all the non-zero matrix entries in *Z* are filled in inadmissible leaves while admissible leaves remain empty because the partial differential operator is local. Hence, the representation of *Z_H_* is exact without approximation.

The resulting structure with a quadtree can be written as

(12) $$Z=\left[\begin{array}{cc} Z_{11} & Z_{12}\\ Z_{21} & Z_{22} \end{array}\right]$$

The process of decomposing the *Z* matrix *LU* of Formula (12) into upper and lower triangular matrices *L* and *U* can be expressed as:

(13) $$Z=LU=\left[\begin{array}{cc} L_{11} & 0\\ L_{21} & L_{22} \end{array}\right]\left[\begin{array}{cc} U_{11} & U_{12}\\ 0 & U_{22} \end{array}\right]=\left[\begin{array}{cc} L_{11}\times U_{11} & L_{11}\times U_{12}\\ L_{21}\times U_{11} & L_{21}\times U_{12}+L_{22}\times U_{22} \end{array}\right]$$

An H-matrix formatted *LU* factorization can be defined by recursively performing Equation (13) and replacing additions and multiplications in it with H-matrix-formatted additions and multiplications (⊕ and ⊗). The H-matrix method can significantly reduce the computational costs of the traditional dense matrix solution method [23,24,25,26]. Once the H-LU factors are obtained, they can be stored, and the H-matrix formatted forward-and-backward substitution (H-FBS) can then be performed very quickly for each RHS vector b in (9). Hence, it exhibits high efficiency for the multiple RHS problems, such as the analysis of the light scattering from multiple incident angles.

### 2.3. Extraction of Light Scattering Characteristics

According to the basic principle of the finite element method, after solving the matrix equations, the equivalent current *J* and the equivalent magnetic current *M* on the surface of the nanostructure can be obtained, and then the scattered electric field and the scattered magnetic field of the nanostructure can be obtained by Formulas (14) and (15).

(14) $$E^{S}=-jw\mu \int_{S}^{}\left[J+\frac{1}{k^{2}}\nabla \left(\nabla^{\prime }\cdot J\right)G\right]ds+\int_{S}^{}M\times \nabla Gds$$

(15) $$H^{S}=-jw\varepsilon \int_{S}^{}\left[M+\frac{1}{k^{2}}\nabla \left(\nabla^{\prime }\cdot M\right)G\right]ds+\int_{S}^{}J\times \nabla Gds$$

where ε,μ are the permittivity and permeability, $k=w\sqrt{\mu \varepsilon }$, $G=e^{-jk\left|r-r^{\prime }\right|}/(4\pi \left|r-r^{\prime }\right|)$, r represents the position of the field point, and $r^{\prime }$ represents the position of the source point.

Regarding the calculation methods of the scattering and absorption cross sections of nanostructures, for the method of moments, it is more suitable to use Poynting’s vector method for calculation. The intensity of scattered light represents the energy scattered by the incident light on the nanostructure, and the calculation formula is as follows:

(16) WS=∮S12Re(Es×Hs*)·ds=∮S12Re(Ss)·ds

Absorbed light intensity represents the energy absorbed by the incident light irradiated on the nanostructure, and the calculation formula is as follows:

(17) Wa=−∮S12Re(E×H*)·ds=−∮S12Re(S)·ds

Here, $W_{a}$ represents the intensity of absorbed light, $W_{S}$ represents the intensity of scattered light, *S* represents the Poynting vector of the total field, and $S^{S}$ represents the Poynting vector of the scattered field.

The scattering cross section represents the ratio of the intensity of scattered light to the intensity of incident light of the nanostructure. The calculation formula is as follows:

(18) $$\sigma_{S}=\frac{W_{S}}{W_{\mathrm{inc}}}$$

The absorption cross section represents the ratio of the absorbed light intensity of the nanostructure to the incident light intensity, and the calculation formula is as follows:

(19) $$\sigma_{a}=\frac{W_{a}}{W_{\mathrm{inc}}}$$

Here, $W_{\mathrm{inc}}$ represents the intensity of incident light.

The extinction cross section (ECS) represents the total amount of scattering and absorption of the incident wave by the nanostructure. It is numerically equal to the sum of the scattering cross section and the absorption cross section. The calculation formula is as follows:

(20) $$\sigma_{e}=\sigma_{a}+\sigma_{s}$$

where $\sigma_{e}$ is the extinction cross section, $\sigma_{a}$ is the absorption cross section, and $\sigma_{s}$ is the scattering cross section.

## 3. Results and Discussion

To demonstrate the accuracy and efficiency of the proposed method, two examples are given. The first example considers a silver nanosphere array, the second example deals with a silver nanocylinder array, and the third example analyzes a gold-nano-truncated cone array. For each example, the ECSs are extracted and compared with the popular commercial software COMSOL to verify the accuracy of the proposed method. The efficiency of the proposed method is verified by comparing its computational costs with those of the traditional MoM. Moreover, this paper explores the variation of the ECS of the nanoarray with the incident angle varying under different wavelengths.

### 3.1. Silver Nanosphere Array

**Figure 4:**
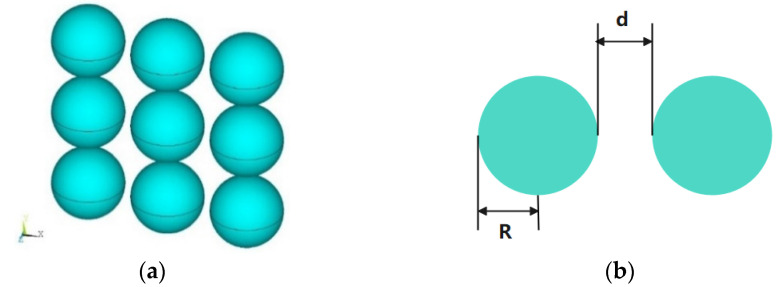
Structure of the silver nanosphere array. (**a**) 3D view; (**b**) dimensions of the nanosphere array, where d = 5 nm, R = 20 nm.

**Figure 5:**
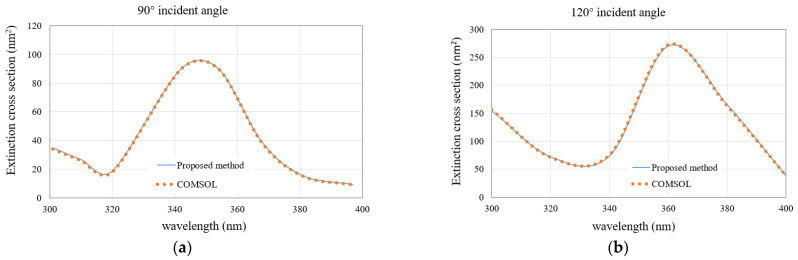
Comparison of the ECS results obtained by the proposed method and the COMSOL software for the nanosphere array. (**a**) 90° incident angle; (**b**) 120° incident angle.

We first analyze a 3 × 3 array of silver nanospheres. Figure 4a,b show the configuration of this nanoarray. The permittivities of silver under different wavelengths are taken from the measured values in [27]. The ECSs of the nanoarray with the light wave illuminates at 90° and 120° incident angles are calculated. The results of the proposed methods are compared with those of the COMSOL software. The triangular patches are used for the MoM discretization, and the minimum mesh size is 3 nm. The wavelength varies from 300 nm to 400 nm. As can be seen from Figure 5a,b, as the incident angle changes from 90° to 120°, the resonance point of the image also changes from 350 nm to 365 nm, and an obvious red shift occurs. Besides, the calculated results are observed to be very consistent with the COMSOL results, which indicates that the proposed method in this paper can accurately calculate the ECSs of the plasmonic nanoarray.

**Figure 6:**
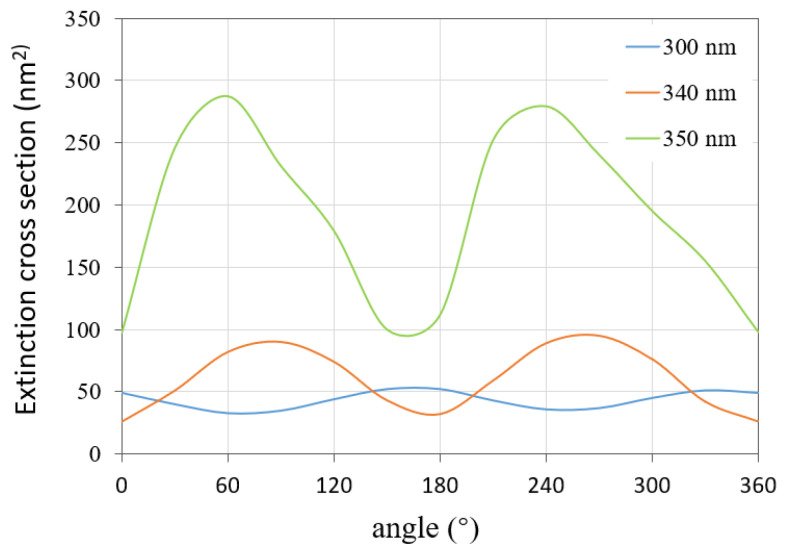
ECSs of the nanosphere array with the incident angle varying from 0° to 360° for different wavelengths.

Then, we calculate the ECS by varying the incident angle from 0° to 360° and fixing the light wavelength at 300 nm, 340 nm, and 350 nm, respectively. The results are shown in Figure 6. It can be seen that, with the variation of incident angle, the ECS of the silver nanosphere array tends to change periodically. Therefore, different incident angles lead to different scattering characteristics, and it is necessary to analyze the light scattering in multiple directions.

**Figure 7:**
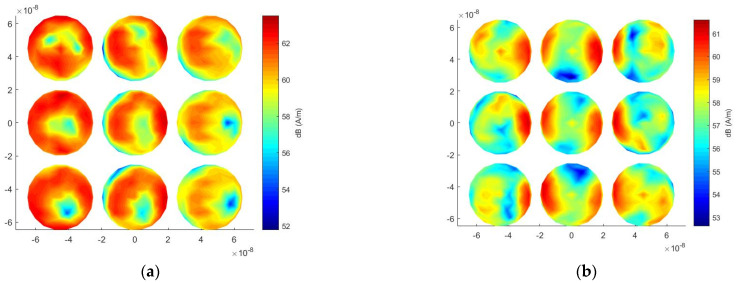
Surface current distribution under different incident angles at 350 nm wavelength for the nanosphere array. (**a**) 120° incident angle; (**b**) 180° incident angle.

Finally, the computational costs of the traditional MoM and the proposed method are reported in Table 1. It can be seen from Table 1 that, for the number of unknowns of 3834, the proposed method only needs 271.6 MB of memory, while the traditional MoM requires 448.6 MB. The proposed method saves 39.4% of memory consumption. For the solution time, the traditional MoM takes 57,009 s because it needs to repeat the iterative calculation for each incident angle, but the proposed method only spends 1351 s, which saves 97.5% computational time. This is because the proposed method only needs to solve the inverse of the matrix once, and then the calculation for each incident angle can be very fast. Therefore, the proposed method is very suitable for the analysis of light scattering in multiple directions. Figure 7a,b show the surface current distributions at a wavelength of 350 nm at an incident angle of 120° and an incident angle of 180°, respectively. In the case of an incident angle of 120°, the closer to the incident wave, the stronger the electric field strength. When the incident angle is 180°, the electric field enhancement between adjacent cells is very significant.

### 3.2. Silver Nanocylinder Array

**Figure 8:**
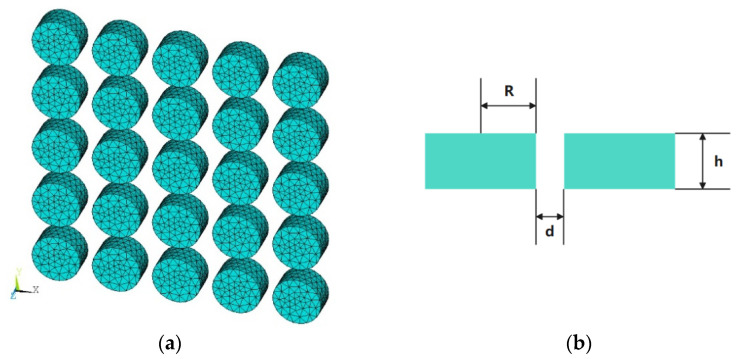
(**a**) 3D view of the silver nanocylinder array with meshes; (**b**) dimensions of the nanocylinder array, where R = 10 nm, d = 5 nm, h = 10 nnm.

**Figure 9:**
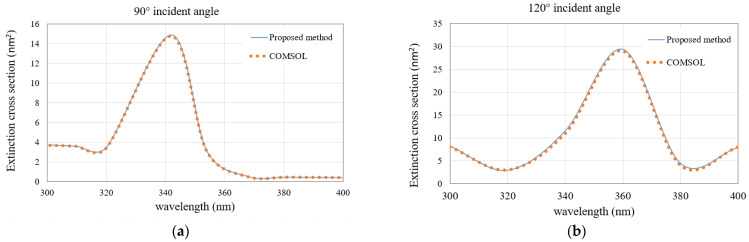
Comparison of the ECS results obtained by the proposed method and the COMSOL software for the nanocylinder array. (**a**) 90° incident angle; (**b**) 120° incident angle.

The second example deals with a 5 × 5 nanocylinders array. The permittivities of silver under different wavelengths are taken from the measured values in [27]. Figure 8a,b show the configuration of this nanoarray. We first calculate the ECS with a wavelength varying from 300 nm to 400 nm by fixing the incident angle of 90° and 120°, respectively. The ECS results of the proposed method and COMSOL are shown in Figure 9a,b. At the incident angle of 90°, the resonance point is close to the wavelength of 340 nm, and when the incident angle increases to 120°, the resonance point is also red shifted close to 360 nm, which is the same as the conclusion of the first example. Again, the calculated results are in good agreement with the COMSOL results.

**Figure 10:**
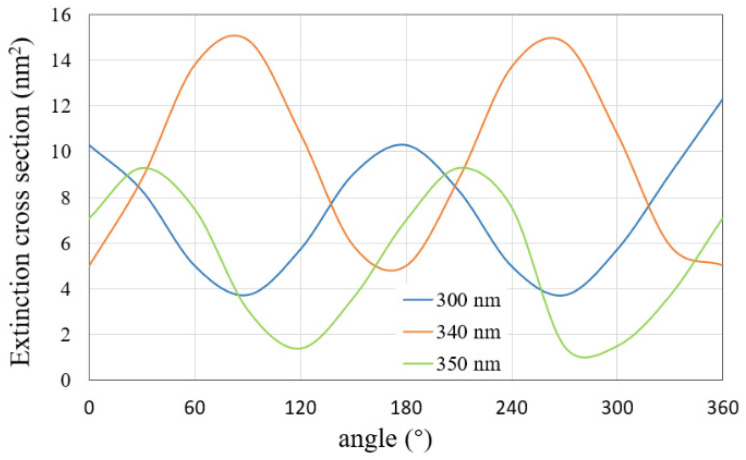
ECSs of the nanocylinder array with the incident angle varying from 0° to 360° for different wavelengths.

Next, we explored the variations of the ECSs of this nanoarray by fixing the incident angle at 300 nm, 340 nm, and 350 nm, respectively. The variation range of the incident angle is from 0° to 360°. The calculation results of the proposed method are shown in Figure 10. The variation of the extinction cross section with the incident angle shows periodic characteristics for each wavelength.

**Figure 11:**
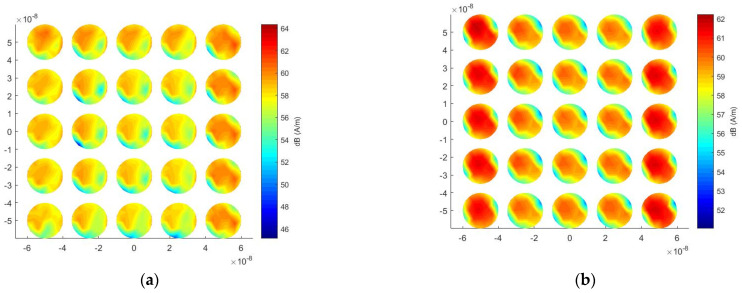
Surface current distribution under different incident angles at 350 nm wavelength for the nanocylinder array. (**a**) 120° incident angle; (**b**) 180° incident angle.

Finally, the efficiency of the proposed method is tested for the calculation of ECSs with multiple incident angles. A number of unknowns of 12,750 are generated after the mesh discretization. For this array, the proposed method saves 97.5% solution time and 47.8% memory usage compared with the traditional MoM, as shown in Table 2. It should be noted that the advantage of the proposed method will be larger and larger as the number of incident angles increases. Figure 11a,b show the surface current distributions at a wavelength of 350 nm at an incident angle of 120° and an incident angle of 180°, respectively.

### 3.3. Gold-Nano-Truncated Cone Array

**Figure 12:**
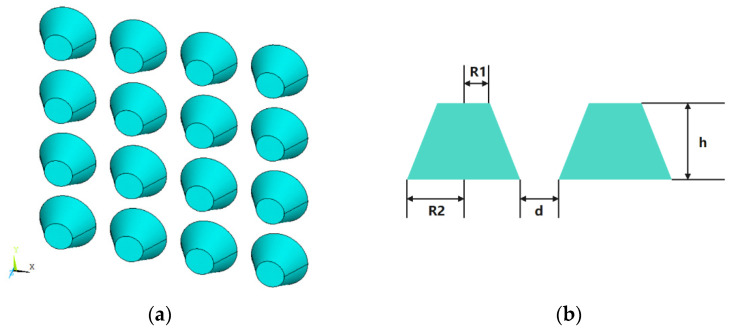
Structure of the gold-nano-truncated cone array. (**a**) 3D view; (**b**) dimensions of the nanosphere array, where d = 10 nm, R2 = 20 nm, R1 = 10 nm, h = 20 nm.

**Figure 13:**
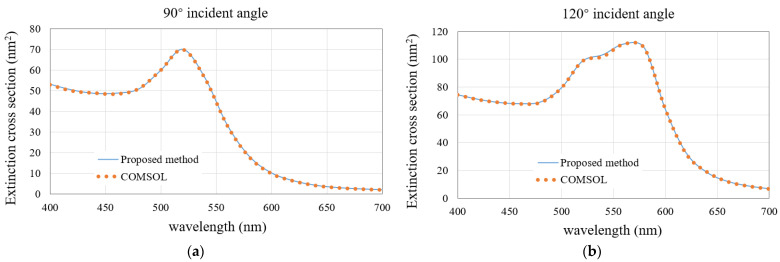
Comparison of the ECS results obtained by the proposed method and the COMSOL software for the nano-truncated cone array. (**a**) 90° incident angle; (**b**) 120° incident angle.

For the third example, a gold-nano-truncated cone array is chosen to demonstrate the generality of the proposed algorithm for a wide range of materials and shapes. The permittivities of gold under different wavelengths are taken from the measured values in [27]. Figure 12a,b show the 3D view of the array and the parameters of a single cell, respectively. We first calculate the ECS with the wavelength varying from 400 nm to 700 nm by fixing the incident angle of 90° and 120°, respectively. The ECS results of the proposed method and COMSOL are shown in Figure 13a,b. When the wavelength changes from 400 nm to 700 nm, the ECS fluctuates significantly. When the incident angle is 90°, the resonance point is located at the wavelength of 520 nm. When the incident angle increases to 120°, the resonance point is red shifted and becomes 560 nm. The calculation results of the method recommended in this paper are consistent with COMSOL.

**Figure 14:**
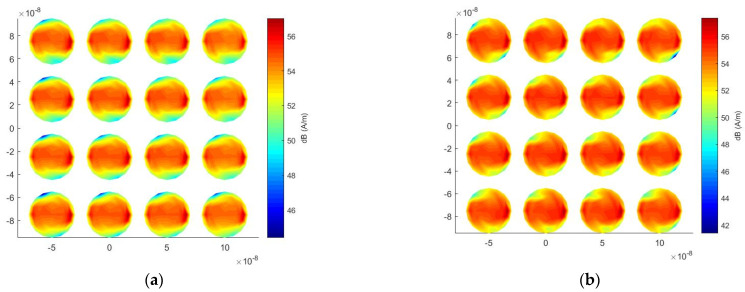
Surface current distribution under different incident angles at 620 nm wavelength for the nano-truncated cone array. (**a**) 120° incident angle; (**b**) 180° incident angle.

Figure 14a,b show the surface-current distributions at a wavelength of 620 nm at an incident angle of 120° and an incident angle of 180°, respectively. In the case of 120°, the field-enhancement phenomenon is more obvious in the region close to the incident wave, and when the incident angle becomes 180°, the surface current distribution is uniform.

## 4. Conclusions

An effective numerical method based on the MoM and the H-matrix method is proposed, which is very suitable for the prediction of light scattering in multiple directions, such as researching the extinction cross sections by changing the incident angle of light. The proposed method directly LU-factorizes the impedance matrix of the MoM and does not require repetitive calculations for each incident angle, which greatly saves prediction time and memory consumption. Examples demonstrate the excellent accuracy and efficiency of the proposed method. It should be noted that the proposed method cannot deal with nanoarrays with a substrate in its current form. However, it can be expanded to the substrate problem by modifying Green’s function, and the H-matrix-based direct method can also be applied.

## Figures and Tables

**Table 1:** Comparison of the computational costs between the proposed method and the traditional MoM for the nanosphere array.

Number of Unknowns	Method	Solution Time (s)	Memory Requirement (MB)
3834	Traditional MoM	57,009.4	448.6
	Proposed method	1351.5	271.6

**Table 2:** Comparison of the computational costs between the proposed method and the traditional MoM for the nanocylinder array.

Number of Unknowns	Method	Solution Time (s)	Memory Requirement (MB)
12,750	Traditional MoM	570,695.6	4961.0
	Proposed method	14,267.4	2591.3
